# Kumar Krishna, in appreciation

**DOI:** 10.3897/zookeys.148.2008

**Published:** 2011-11-21

**Authors:** Michael S. Engel, David A. Grimaldi

**Affiliations:** 1 Division of Entomology, Natural History Museum, and Department of Ecology & Evolutionary Biology, 1501 Crestline Drive – Suite 140, University of Kansas, Lawrence, Kansas 66049-2811, USA; 2 Division of Invertebrate Zoology, American Museum of Natural History, Central Park West at 79th Street, New York, New York 10024-5192, USA

It is with admiration and fondness that we dedicate this special issue of *ZooKeys* to Professor Kumar Krishna, dean of Isoptera research. This collection of papers is a humble testament of appreciation by the various biologists who are dedicated to studying termites, insects that are popularly maligned but actually of profound behavioral and ecological importance. Kumar’s influence extends beyond the scope of isopterological studies and so several of the papers included herein are from contributors on other lineages of insects who have been similarly inspired by his indomitable spirit.

Kumar grew up in Dehra Dun in northern India, in the foothills of the Himalayas. His father was a physician, and was also one of the first Indians to be commissioned in the British army, in fact serving as a major in World War I. Kumar went to Agra University, earning a Bachelor of Science in 1950, and shortly thereafter a Master of Science degree from Lucknow University in 1952. He then served as a Research Assistant (1952–1954) to Mittan Lal Roonwal (1908–1990) at the Forest Research Institute in Dehra Dun, where he developed his interest in Isoptera. Roonwal is well known among Isoptera workers for his comprehensive papers on the systematics and general biology of termites from India and surrounding areas. Immediately thereafter, he moved to the U.S. where he was a graduate student and employed as a research assistant at the University of Minnesota from 1954–55. It was during this time that he wrote to Prof. Alfred E. Emerson (1896–1976) about graduate studies, and was soon thereafter accepted into Emerson’s lab. Emerson was a professor at the University of Chicago from 1929–1962 and well known as the leading authority on the systematics and general evolution of termites. He was also a coauthor of the classic *Principles of Animal Ecology* (Allee et al. 1949), and a colleague of William Morton Wheeler (1865–1937), the authority on ants at Harvard University and predecessor of E.O. Wilson. It was Emerson and Wheeler who promoted the concept of an insect colony as a superorganism, and both men built massive, global collections of their research groups. Emerson was also a colleague of the architects of the New Synthesis in evolutionary biology – Ernst Mayr (1904–2005), George Gaylord Simpson (1902–1984), Theodosius Dobzhansky (1900–1975), and G. [George] Ledyard Stebbins (1906–2000) – as well as president of several important scientific societies, and a member of the prestigious National Academy of Science. He was hugely influential, and he had a lasting impact on Kumar.

**Figure:**
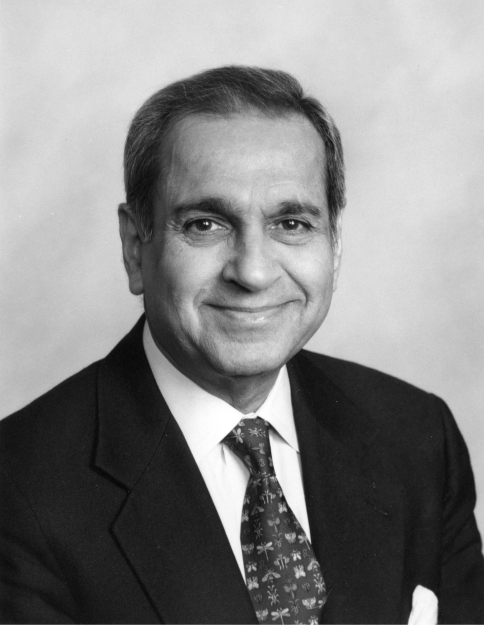
**Figure 1.** Prof. Dr. Kumar Krishna.

At the University of Chicago, Kumar began a comprehensive revision of the world genera of drywood termites, still the major reference on the family Kalotermitidae (Krishna 1961). It was there that Kumar met his lifelong partner, Valerie. Valerie was studying English Literature and worked at the University of Chicago Press as an editor and proofreader, a skill that would be thoroughly utilized later on. Kumar and Valerie married in 1960. Kumar completed his doctorate in Evolutionary Biology from the University of Chicago in 1961, and was employed as a National Science Foundation postdoctoral fellow there until 1962 when he and Valerie departed for New York, and where Emerson donated his massive collection of termites to the American Museum of Natural History (AMNH). Kumar became an instructor at City College of the City University of New York (CUNY) in 1962, and a Research Associate in residence in the AMNH’s Department of Entomology (now the Division of Invertebrate Zoology). He became Assistant Professor in 1964, Associate Professor in 1969, and full Professor in 1973, principally teaching Biology, Entomology, and Evolutionary Biology. Valerie herself was a Professor of English at CCNY, whose particular interest was Chaucer and *The Canterbury Tales*, and Malory’s *Le Morte D’Arthur*. Though it took time away from research, Kumar held many important administrative posts at CCNY, which had significant impact on City College, CUNY, and the biological sciences. He served as Chair of the Department of Biology (1963–1968), deputy chair for the Department of Biology (1972–1975, 1978–1981), as Chair of the Graduate Program in Biology (1972–1974, 1978–1981), a member of the Faculty Research Award Program (1978–1981, 1985–1993), and as a member of the University Committee on Research (1981–1983, 1994–1996). There were some very challenging times during this period. In the 1950’s, City College was a crucible of intellectualism, but was overcome by student radicalism in the 1960’s. In the 1970’s, New York suffered a severe financial crisis that cut deeply into the budgets of CUNY and other city organizations. But, biology prospered at CCNY, and the department even embarked on a symbiotic program with the AMNH in graduate student training in systematics.

Despite Kumar’s administrative and teaching loads, perhaps the most impressive achievements are that he continued to produce influential research, funded by National Science Foundation research grants. It was during this time, for example, that Kumar Krishna and Frances M. Weesner organized and contributed to the seminal, two-volume work, *Biology of the Termites* (1969–70), synthesizing all major topics on termite biology and systematics. Interestingly, publication of *Biology of the Termites* coincided with the first major book by E.O. Wilson, *The Insect Societies* (1971). It was the heyday of social insects. Although two recent volumes on termite biology have been produced (Abe et al. 2000; Bignell et al. 2010), Krishna and Weesner remains an invaluable reference. On far more adventurous fronts, Kumar and Valerie made numerous (sometimes dangerous) expeditions to collect termite specimens. Of particularly note are four weeks collecting in Myanmar (1961); a ten-month expedition across Sri Lanka, India, Thailand, and Taiwan (1968–1969); eight weeks collecting in Borneo, Malaya, and Singapore (1977–1978); four weeks in Borneo (1984–1985); four weeks in Sumatra (1988); three weeks in Malaya (1990); and seven weeks in Sulawesi and Java (1992). When Kumar retired in 1996 he was appointed Emeritus Professor at CCNY and took up residence full time on the fifth floor at the AMNH, devoting his efforts to the world’s largest and most comprehensive termite collection, and to research.

**Figure:**
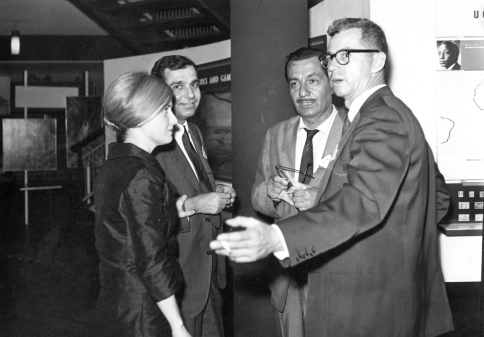
**Figure 2.** Valerie Krishna, Kumar Krishna, Renato Araujo (1912–1978), and Alexander Sokoloff (1920–2011) at the XII International Congress of Entomology, London, 1964.

It is safe to say that no one today has such comprehensive knowledge of termites globally, particularly their systematics, taxonomy, morphology, biogeography, and the fossil record. When Emerson was working, for example, there was only one Cretaceous termite known, *Cretatermes carpenteri* Emerson. Now, there are 36 species and 32 genera, nearly a quarter of which we have had the pleasure of working with Kumar to describe, but more importantly he has united a critical study of fossil termites with that of their modern counterparts. Emerson would have been immensely pleased to see how much more we now know about termite diversity, relationships, and evolution, largely as a result of Kumar’s efforts. Kumar’s encyclopedic knowledge makes him the ideal person to have been the principal author of the upcoming and highly anticipated magnum opus, *Treatise on the Isoptera of the World* (2012). At 2400 single-spaced manuscript pages, the work is immense, 85% of which is a taxonomic compendium of the 3138 living and fossil termite species of the world (as of 26 March 2011) – incorporating a plethora of nomenclatural corrections made along the way, all based on direct study of over 4000 original taxonomic references and the more significant biological ones. Whereas many catalogues simply add to previous ones, propagating errors, this work was created de novo. Valerie applied her editorial acumen, spending months fastidiously proofing the manuscript for style and punctuation, a testament to the regard she and Kumar hold for each other.

Putting his considerable academic accomplishments aside, Kumar is one of the most positive, jovial, and generous individuals we know. Now in his eighties, his enthusiasm and energy for termite research seem tireless. When he worked on the Termitidae in Miocene Dominican amber, for example, Kumar spent hours each day hunched over his microscope, examining, sorting, comparing, and measuring hundreds of specimens and describing dozens of species. He worked like a graduate student. Indeed, there is perhaps no more enjoyable way for us to spend an afternoon than taking turns peering into a microscope alongside Kumar, discussing the details of some challenging fossil or exotic living specimen. We look forward to his residence on the fifth floor of the museum for many years to come, where we can peer through the microscope together in excited discussion.

## Publications of Kumar Krishna

### Completed works as of 5 September 2011

1. Roonwal ML, Krishna K (1955) Systematics of Oriental termites. II. A new species, *Coptotermes guarii*, from Ceylon. Indian Journal of Agricultural Science 25(2): 143–152.

2. Krishna K (1956) Two new species of *Coptotermes* Wasmann from Malaya (Isoptera, Rhinotermitidae, Coptotermitinae). American Museum Novitates 1809: 1–5.

3. Krishna K (1960) Social insects. In: McGraw-Hill Encyclopedia of Science and Technology [Volume 14]. McGraw-Hill, New York, NY, 405–407.

4. Krishna K (1961) A generic revision and phylogenetic study of the family Kalotermitidae (Isoptera). Bulletin of the American Museum of Natural History 122(4): 303–408.

5. Krishna K, Emerson AE (1962) New species of the genus *Glyptotermes* Froggatt from the Papuan, Oriental, Ethiopian, and Neotropical Regions (Isoptera, Kalotermitidae). American Museum Novitates 2089: 1–65.

6. Krishna K (1962) New species and a hitherto undescribed imago caste of the genus *Calcaritermes* Snyder (Isoptera, Kalotermitidae). American Museum Novitates 2098: 1–13.

7. Krishna K (1962) New species of the genera *Allotermes* Wasmann, *Bicornitermes* Krishna, *Epicalotermes* Silvestri, and *Procryptotermes* Holmgren (Isoptera, Kalotermitidae). American Museum Novitates 2119: 1–25.

8. Krishna K (1963) The evolution of the family Kalotermitidae, Isoptera. Symposia Genetica et Biologica Italica [Atti IV Congresso U.I.E.I.S., Pavia, 9–14 September 1961] 11: 202–209.

9. Krishna K (1963) The African genus *Foraminitermes* Holmgren (Isoptera, Termitidae, Termitinae). American Museum Novitates 2161: 1–23.

10. Krishna K (1963) A systematic catalogue of the main identified entomological collection at the Forest Research Institute, Dehradun, Part 32, Order Lepidoptera, Family Papilionidae. Indian Forest Leaflet 121(4): 396–408.

11. Krishna K (1965) A new species of termite from the Nicobar Islands (Isoptera, Termitidae, Nasutitermitinae). Entomologiske Meddelelser 34: 107–109.

12. Krishna K (1965) Termites (Isoptera) of Burma. American Museum Novitates 2210: 1–34.

13. Krishna K (1966) Key to eight termite genera: *Kalotermes*, *Incisitermes*, *Cryptotermes*, *Zootermopsis*, *Heterotermes*, *Reticulitermes*, *Coptotermes*, and *Amitermes*. Cooperative Economic Insect Report, Plant Pest Control Division, Agricultural Research Service, United States Department of Agriculture 16(47): 1091–1098.

14. Krishna K, Araujo RL (1968) A revision of the neotropical termite genus *Neocapritermes* (Isoptera, Termitidae, Termitinae). Bulletin of the American Museum of Natural History 138(3): 83–130.

15. Krishna K (1968) Phylogeny and generic reclassification of the *Capritermes* complex (Isoptera, Termitidae, Termitinae). Bulletin of the American Museum of Natural History 138(5): 261–324.

16. Krishna K, Weesner FM (1969) Biology of Termites [Volume I]. Academic Press, New York, NY, xiii+598 pp.

17. Krishna K (1969) Introduction. In: Krishna K, Weesner FM (Eds) Biology of Termites [Volume I]. Academic Press, New York, NY, 1–17 [total pages xiii+598 pp.].

18. Krishna K, Weesner FM (1970) Biology of Termites [Volume II]. Academic Press, New York, NY, xiv+[i]+643 pp.

19. Krishna K (1970) Taxonomy, phylogeny, and distribution of termites. In: Krishna K, Weesner FM (Eds) Biology of Termites [Volume II]. Academic Press, New York, NY, 127–152 [total pages xiv+[i]+643 pp.].

20. Krishna K (1972) A revision of the Indomalayan termite genus *Homallotermes* (Isoptera, Termitidae, Termitinae). American Museum Novitates 2489: 1–23.

21. Krishna K (1974) Isoptera. In: Preece WE (Ed) Encyclopaedia Britannica [15^th^ Edition, Volume 9]. Encyclopaedia Britannica, Inc., London, UK, 1049–1054.

22. Emerson AE, Krishna K (1975) The termite family Serritermitidae (Isoptera). American Museum Novitates 2570: 1–31.

23. Krishna K, Adams C (1982) The Oriental termite genus *Labritermes* Holmgren (Isoptera, Termitidae, Termitinae). American Museum Novitates 2735: 1–14.

24. Krishna K, Emerson AE (1983) A new fossil species of termite from Mexican amber, *Mastotermes electromexicus* (Isoptera, Mastotermitidae). American Museum Novitates 2767: 1–8.

25. Krishna K, Bacchus S (1987) A new fossil species of termite from Dominican amber, *Cryptotermes yamini* (Isoptera, Kalotermitidae). American Museum Novitates 2884: 1–5.

26. Krishna K (1989) Order Isóptera: Termites. In: Borror DJ, Triplehorn CA, Johnson NF (Eds) An Introduction to the Study of Insects [6^th^ Edition]. Saunders College Publishing, Philadelphia, PA, 234–241 [xiv+875 pp.].

27. Krishna K (1990) Isoptera. Bulletin of the American Museum of Natural History 195: 76–81. [Grimaldi DA (Ed) Insects from the Santana Formation, Lower Cretaceous, of Brazil. Bulletin of the American Museum of Natural History 195: 1–191].

28. Krishna K, Grimaldi D (1991) A new fossil species from Dominican amber of the living Australian termite genus *Mastotermes* (Isoptera: Mastotermitidae). American Museum Novitates 3021: 1–10.

29. Krishna K (1992) Isoptera. In: McHenry R (Ed) The New Encyclopaedia Britannica [15^th^ Edition, Volume 21, Macropaedia]. Encyclopaedia Britannica, Inc., Chicago, IL, 675–679 [total volume pages [iii]+1016 pp.].

30. Krishna K (1996) New fossil species of termites of the subfamily Nasutitermitinae from Dominican and Mexican amber (Isoptera, Termitidae). American Museum Novitates 3176: 1–13.

31. Thorne BL, Grimaldi DA, Krishna K (2000) Early fossil history of the termites. In: Abe T, Bignell DE, Higashi M (Eds) Termites: Evolution, Sociality, Symbioses, Ecology. Kluwer Academic Publishers, Dordrecht, The Netherlands, 77–93 [total volume pages xxii+466 pp.].

32. Krishna K, Grimaldi D (2000) A new subfamily, genus, and species of termite (Isoptera) from New Jersey Cretaceous amber. In: Grimaldi D (Ed) Studies on Fossils in Amber, with Particular Reference to the Cretaceous of New Jersey. Backhuys Publishers, Leiden, The Netherlands, 133–140 [total volume pages viii+498 pp.].

33. Engel MS, Krishna K (2001) *Kalotermes* Hagen, 1853 (Insecta, Isoptera): Proposed designation of *Termes flavicollis* Fabricius, 1793 as the type species. Bulletin of Zoological Nomenclature 58(2): 100–104.

34. Engel MS, Krishna K (2001) Macrotermitinae Kemner, 1934 (Insecta, Isoptera): Proposed precedence over Acanthotermitinae Sjöstedt, 1926. Bulletin of Zoological Nomenclature 58(3): 206–209.

35. Krishna K (2001) Southeast Asian species of the genus *Dicuspiditermes* (Isoptera: Termitidae: Termitinae). Sociobiology 37(3A): 399–488.

36. Engel MS, Krishna K (2002) *Cryptotermes dudleyi* Banks, 1918 (Insecta, Isoptera): Proposed precedence over *Calotermes* (*Cryptotermes*) *jacobsoni* Holmgren, 1913. Bulletin of Zoological Nomenclature 59(2): 90–92.

37. Krishna K, Grimaldi DA (2003) The first Cretaceous Rhinotermitidae (Isoptera): A new species, genus, and subfamily in Burmese amber. American Museum Novitates 3390: 1–10.

38. Krishna K (2003) A new species, *Cavitermes rozeni* (Isoptera: Termitidae: Termitinae), from Brazil. Journal of the Kansas Entomological Society 76(2): 92–95.

39. Engel MS, Krishna K, Boyko C (2003) Termopsidae Holmgren, 1911, *Termopsis* Heer, 1849 and *Miotermes* Rosen, 1913 (Insecta, Isoptera): Proposed conservation of prevailing usage by the designation of *Termes bremii* Heer, 1849 as the type species of *Termopsis*. Bulletin of Zoological Nomenclature 60(2): 119–123.

40. Engel MS, Krishna K (2004) Family-group names for termites (Isoptera). American Museum Novitates 3432: 1–9.

41. Engel MS, Krishna K (2004) Comment on the proposed conservation of prevailing usage of Termopsidae Holmgren, 1911, *Termopsis* Heer, 1849 and *Miotermes* Rosen, 1913 (Insecta, Isoptera). Bulletin of Zoological Nomenclature 61(3): 169–170.

42. Engel MS, Krishna K (2004) An overlooked family-group name for termites (Isoptera). Entomological News 115(3): 168.

43. Engel MS, Krishna K (2005) *Nasutitermes* Dudley, 1890, *Microcerotermes* Silvestri, 1901 and Nasutitermitinae Hare, 1937 (Insecta, Isoptera): Proposed conservation. Bulletin of Zoological Nomenclature 62(1): 8–13.

44. Engel MS, Krishna K (2005) Comment on the proposed suppression of *Eutermes* Heer, 1849 to conserve the generic names *Nasutitermes* Dudley, 1890 and *Microcerotermes* Silvestri, 1901, and on the proposed designation of *Eutermes costalis* Holmgren, 1910 as type species of *Nasutitermes* Dudley, 1890 (Insecta, Isoptera). Bulletin of Zoological Nomenclature 62(4): 240.

45. Krishna K (2005) Order Isoptera: Termites. In: Triplehorn CA, Johnson NF (Ed) Borror and DeLong’s Introduction to the Study of Insects [7^th^ Edition]. Brooks/Cole, Belmont, CA, 252–259 [total volume pages x+864 pp.].

46. Engel MS, Krishna K (2007) Two overlooked family-group names for fossil termites (Isoptera: Mastotermitidae). Entomological News 118(1): 105–106.

47. Lo N, Engel MS, Cameron S, Nalepa CA, Tokuda G, Grimaldi D, Kitade O, Krishna K, Klass K-D, Maekawa K, Miura T, Thompson GJ (2007) Spare Isoptera: A comment on Inward et al. Biology Letters 3(5): 562–563.

48. Engel MS, Krishna K (2007) New *Dolichorhinotermes* from Ecuador and in Mexican amber (Isoptera: Rhinotermitidae). American Museum Novitates 3592: 1–8.

49. Engel MS, Krishna K (2007) Drywood termites in Dominican amber (Isoptera: Kalotermitidae). Beiträge zur Entomologie 57(2): 263–275.

50. Engel MS, Krishna K (2007) *Reticulitermes* Holmgren, 1913 (Insecta, Isoptera): Proposed precedence over *Maresa* Giebel, 1856. Bulletin of Zoological Nomenclature 64(4): 230–234.

51. Engel MS, Grimaldi DA, Krishna K (2007) Primitive termites from the Early Cretaceous of Asia (Isoptera). Stuttgarter Beiträge zur Naturkunde, Serie B, Geologie und Paläontologie 371: 1–32.

52. Engel MS, Grimaldi DA, Krishna K (2007) A synopsis of Baltic amber termites (Isoptera). Stuttgarter Beiträge zur Naturkunde, Serie B, Geologie und Paläontologie 372: 1–20.

53. Grimaldi DA, Engel MS, Krishna K (2008) The species of Isoptera (Insecta) from the Early Cretaceous Crato Formation: A revision. American Museum Novitates 3626: 1–30.

54. Krishna K, Grimaldi DA (2009) Diverse Rhinotermitidae and Termitidae (Isoptera) in Dominican amber. American Museum Novitates 3640: 1–48.

55. Engel MS, Grimaldi DA, Krishna K (2009) Termites (Isoptera): Their phylogeny, classification, and rise to ecological dominance. American Museum Novitates 3650: 1–27.

56. Krishna K, Engel MS (2011) *Cryptotermes dudleyi* Banks, 1918 (Insecta, Isoptera): Proposed precedence over *Calotermes havilandi parasita* Wasmann, 1910 (currently *Cryptotermes parasita*). Bulletin of Zoological Nomenclature 68(2): 109–112.

57. Krishna K, Engel MS, Grimaldi DA, Krishna V (2012) Treatise on the Isoptera of the world. Bulletin of the American Museum of Natural History. In press.

## Taxa proposed by Kumar Krishna

### Available names as of 5 September 2011 Unranked names (2):

Euisoptera Engel, Grimaldi, & Krishna

Neoisoptera Engel, Grimaldi, & Krishna

### Family-group names (6):

†Archeorhinotermitinae Krishna & Grimaldi

Archotermopsidae Engel, Grimaldi, & Krishna

†Carinatermitinae Krishna & Grimaldi

†Cratomastotermitidae Engel, Grimaldi, & Krishna

Sphaerotermitinae Engel & Krishna

Syntermitinae Engel & Krishna

### Genus-group names (19):

†*Archeorhinotermes* Krishna & Grimaldi

†*Baissatermes* Engel, Grimaldi, & Krishna

*Bicornitermes* Krishna

*Bifiditermes* Krishna

*Ceratokalotermes* Krishna

*Comatermes* Krishna

†*Dharmatermes* Engel, Grimaldi, & Krishna

*Dicuspiditermes* Krishna

†*Garmitermes* Engel, Grimaldi, & Krishna

*Incisitermes* Krishna

†*Kachinitermes* Engel, Grimaldi, & Krishna

†*Khanitermes* Engel, Grimaldi, & Krishna

*Labiocapritermes* Krishna

*Marginitermes* Krishna

†*Melqartitermes* Engel, Grimaldi, & Krishna

†*Mylacrotermes* Engel, Grimaldi, & Krishna

*Postelectrotermes* Krishna

†*Tanytermes* Engel, Grimaldi, & Krishna

*Tauritermes* Krishna

### Species-group names (106):

*Aciculitermes maymyoensis* Krishna

*Allotermes denticulatus* Krishna

*Allotermes papillifer* Krishna

†*Amitermes lucidus* Krishna & Grimaldi

*Angulitermes paanensis* Krishna

*Angulitermes resimus* Krishna

†*Anoplotermes bohio* Krishna & Grimaldi

†*Anoplotermes cacique* Krishna & Grimaldi

†*Anoplotermes carib* Krishna & Grimaldi

†*Anoplotermes maboya* Krishna & Grimaldi

†*Anoplotermes naboria* Krishna & Grimaldi

†*Anoplotermes nitaino* Krishna & Grimaldi

†*Anoplotermes quisqueya* Krishna & Grimaldi

†*Anoplotermes taino* Krishna & Grimaldi

†*Archeorhinotermes rossi* Krishna & Grimaldi

†*Atlantitermes antillea* Krishna & Grimaldi

†*Atlantitermes caribea* Krishna & Grimaldi

†*Atlantitermes magnoculus* Krishna & Grimaldi

†*Baissatermes lapideus* Engel, Grimaldi, & Krishna

*Bicornitermes bicornis* Krishna

*Bicornitermes emersoni* Krishna

*Bulbitermes prabhae* Krishna

*Calcaritermes colei* Krishna

*Calcaritermes rioensis* Krishna

*Calcaritermes snyderi* Krishna

†*Caribitermes hispaniola* Krishna & Grimaldi

*Cavitermes rozeni* Krishna

†*Constrictotermes electroconstrictus* Krishna

*Coptotermes bentongensis* Krishna

†*Coptotermes hirsutus* Krishna & Grimaldi

†*Coptotermes paleodominicanus* Krishna & Grimaldi

*Coptotermes sepangensis* Krishna

†*Cryptotermes yamini* Krishna & Bacchus

†*Cryptotermes glaesarius* Engel & Krishna

†*Dharmatermes avernalis* Engel, Grimaldi, & Krishna

*Dicuspiditermes cacuminatus* Krishna

*Dicuspiditermes fissifex* Krishna

*Dicuspiditermes kistneri* Krishna

*Dicuspiditermes spinitibialis* Krishna

†*Dolichorhinotermes apopnus* Engel & Krishna

*Dolichorhinotermes lanciarius* Engel & Krishna

*Epicalotermes planifrons* Krishna

*Foraminitermes coatoni* Krishna

*Foraminitermes harrisi* Krishna

†*Garmitermes succineus* Engel, Grimaldi, & Krishna

*Glyptotermes adamsoni* Krishna & Emerson

*Glyptotermes concavifrons* Krishna & Emerson

†*Glyptotermes grimaldii* Engel & Krishna

*Glyptotermes guamensis* Krishna & Emerson

*Glyptotermes hendrickxi* Krishna & Emerson

*Glyptotermes jurioni* Krishna & Emerson

*Glyptotermes kirbyi* Krishna & Emerson

*Glyptotermes lighti* Krishna & Emerson

*Glyptotermes longipennis* Krishna & Emerson

*Glyptotermes longuisculus* Krishna & Emerson

*Glyptotermes nissanensis* Krishna & Emerson

*Glyptotermes palauensis* Krishna & Emerson

†*Glyptotermes paleoliberatus* Engel & Krishna

*Glyptotermes parki* Krishna & Emerson

*Glyptotermes parvoculatus* Krishna & Emerson

*Glyptotermes rotundifrons* Krishna & Emerson

*Glyptotermes schmidti* Krishna & Emerson

*Glyptotermes seeversi* Krishna & Emerson

*Glyptotermes sicki* Krishna & Emerson

*Glyptotermes sinomalatus* Krishna & Emerson

*Glyptotermes truncatus* Krishna & Emerson

*Glyptotermes tuberifer* Krishna & Emerson

*Homallotermes eleanorae* Krishna

*Homallotermes exiguus* Krishna

†*Incisitermes peritus* Engel & Krishna

*Labritermes emersoni* Krishna & Adams

*Labritermes kistneri* Krishna & Adams

†*Mastotermes electrodominicus* Krishna & Grimaldi

†*Mastotermes electromexicus* Krishna & Emerson

†*Meiatermes araripena* Krishna

†*Melqartitermes myrrheus* Engel, Grimaldi, & Krishna

†*Microcerotermes insulanus* Krishna & Grimaldi

†*Microcerotermes setosus* Krishna & Grimaldi

*Microcerotermes uncatus* Krishna

*Mirocapritermes valeriae* Krishna

†*Mylacrotermes cordatus* Engel, Grimaldi, & Krishna

†*Nasutitermes amplioculatus* Krishna & Grimaldi

†*Nasutitermes electrinus* Krishna

†*Nasutitermes electronasutus* Krishna

*Nasutitermes fabricii* Krishna

†*Nasutitermes incisus* Krishna & Grimaldi

†*Nasutitermes magnocellus* Krishna & Grimaldi

†*Nasutitermes medioculatus* Krishna & Grimaldi

†*Nasutitermes pilosus* Krishna & Grimaldi

†*Nasutitermes rotundicephalus* Krishna & Grimaldi

†*Nasutitermes seminudus* Krishna & Grimaldi

*Neocapritermes araguaia* Krishna & Araujo

*Neocapritermes guyana* Krishna & Araujo

*Neocapritermes talpoides* Krishna & Araujo

*Neocapritermes taracua* Krishna & Araujo

*Neocapritermes utiariti* Krishna & Araujo

†*Parvitermes longinasus* Krishna & Grimaldi

*Procapritermes parvulus* Krishna

*Procryptotermes falcifer* Krishna

†*Proelectrotermes holmgreni* Engel, Grimaldi, & Krishna

†*Subulitermes hispaniola* Krishna & Grimaldi

†*Subulitermes insularis* Krishna & Grimaldi

†*Tanytermes anawrahtai* Engel, Grimaldi, & Krishna

†*Termes primitivus* Krishna & Grimaldi

†*Termopsis ukapirmasi* Engel, Grimaldi, & Krishna

†*Velocitermes bulbus* Krishna & Grimaldi
